# Isolation of Ovicidal Fungi from Fecal Samples of Captive Animals Maintained in a Zoological Park

**DOI:** 10.3390/jof3020029

**Published:** 2017-06-02

**Authors:** José A. Hernández, Rosa A. Vázquez-Ruiz, Cristiana F. Cazapal-Monteiro, Esther Valderrábano, Fabián L. Arroyo, Iván Francisco, Silvia Miguélez, Rita Sánchez-Andrade, Adolfo Paz-Silva, María S. Arias

**Affiliations:** 1 COPAR (Control of Parasites), Animal Pathology Department, Veterinary Faculty, Santiago de Compostela University, Campus Universitario, 27002 Lugo, Spain; joseangelher@gmail.com (J.A.H.); cristianafcm@gmail.com (C.F.C.-M.); leonardoarroba@hotmail.com (F.L.A.); ivanfranciscovazquez@gmail.com (I.F.); silvia.miguelez.r@gmail.com (S.M.); rita.sanchez-andrade@usc.es (R.S.-A.); adolfo.paz@usc.es (A.P.-S.); 2 Department of Botany, Veterinary Faculty, Santiago de Compostela University, Campus Universitario, 27002 Lugo, Spain; rosana.vazquez@usc.es; 3 Marcelle Natureza Zoological Park, Outeiro de Rei, 27122 Lugo, Spain; servet.marcellenatureza@gmail.com

**Keywords:** ovicidal fungi, zoological park, biological control, sustainability

## Abstract

There are certain saprophytic fungi in the soil able to develop an antagonistic effect against eggs of parasites. Some of these fungal species are ingested by animals during grazing, and survive in their feces after passing through the digestive tract. To identify and isolate ovicidal fungi in the feces of wild captive animals, a total of 60 fecal samples were taken from different wild animals kept captive in the Marcelle Natureza Zoological Park (Lugo, Spain). After the serial culture of the feces onto Petri dishes with different media, their parasicitide activity was assayed against eggs of trematodes (*Calicophoron daubneyi*) and ascarids (*Parascaris equorum*). Seven fungal genera were identified in the feces. Isolates from *Fusarium*, *Lecanicillium*, *Mucor*, *Trichoderma*, and *Verticillium* showed an ovicidal effect classified as type 3, because of their ability to adhere to the eggshell, penetrate, and damage permanently the inner embryo. *Penicillium* and *Gliocladium* developed a type 1 effect (hyphae attach to the eggshell but morphological damage was not provoked). These results provide very interesting and useful information about fungi susceptible for being used in biological control procedures against parasites.

## 1. Introduction

Adult stages of certain helminths affecting animals release eggs that are passed out in the feces. Once in the soil, different phases are accomplished to attain the infective stage, and the life-cycle is completed when animals feed on pastures [1]. Some of these helminths are zoonotic agents because they can infect humans also [2].

In the soil, there are several possibilities for the transmission of helminths through eggs shed in feces: (1) a larva originates inside the egg in the soil, but the larva does not exit from the egg until it is ingested by the host and excysts at the gut level (nematodes: ascarids, trichurids); (2) the larva originates in the egg, leaves it, and molts in the environment until the infective stage is reached (nematodes: strongylids, ancylostomids), or (3) the larva abandons the egg and infects an intermediate host to reach the infective stage (trematodes, cestodes) [3,4]. In terms of moving capability, parasites remain immobile and confined in the eggs until they are ingested (ascarids, trichurids), or leave actively the eggs and scroll in the environment (trematodes; nematodes: strongylids, ancylostomids).

As occurs with domestic species, wild captive animals maintained always in the same paddock (continuous grazing) can be at risk of infection by certain helminths, because they are constantly shedding eggs to the environment. Despite the administration of successful therapy based on anthelmintics, these animals infect again because of the ingestion of infective stages when feeding on grass [5].

Under natural conditions, the presence of some saprophytic fungi in soil that can develop antagonistic effects on the eggs of parasites, with the aim to take nutrients as C or N, has been reported [6]. The ability of some of these fungi to pass through the gastrointestinal tract of different animal species and survive in their feces has been previously reported, concerning mainly *Duddingtonia flagrans*, *Pochonia chlamydosporia*, or *Mucor circinelloides* [2,7,8,9]. Hence, their employment has been notably advised in the last decades as a contribution to the control of parasites affecting livestock.

Studies performed on different countries demonstrated the presence of nematophagous fungi in fecal samples from domestic animal species [4,10,11,12,13,14,15,16,17].

Most known species with ovicidal activity are *Verticillium* spp., *Pochonia chlamydosporia*, *Paecilomyces lilacinus*, *Trichoderma* spp., or *Mucor circinelloides* [18,19,20]. By developing the phases of adhesion, colonization, penetration, and deliberation, these fungi develop an ovicidal activity [21,22]. Recently, the role of *Trichoderma* spp. in the biological control of insects pest such us *Xylotrechus arvicola* and *Acanthoscelides obtectus* has been described [23,24]. The objective of this study was to evaluate the presence of fungi with ovicidal activity in the feces of wild animals maintained captive at the “Marcelle Natureza” zoological park (NW Spain).

## 2. Material and Methods

### 2.1. Marcelle Natureza Zoological Park

The current investigation was conducted in “Marcelle Natureza”, a 20 ha zoological park located in NW Spain (Outeiro de Rei, Lugo) (43°4′4.71′′ N, 7°37′53.50′′ W). Collection animals live in fenced semi-free ranging parcels of various sizes. The animals are routinely dewormed in spring and autumn by adding granulated anthelmintic preparations to their diet. Removal of fecal material is performed daily in the paddocks by the keepers, before the visitors arrive.

### 2.2. Collection and Analysis of Fecal Samples

Freshly deposited feces were taken in the morning from a total of 60 paddocks, then put into plastic flasks, and finally brought to the lab. Each fecal sample was analyzed by the flotation test to determine the presence of coccidian cysts/oocysts, eggs of cestodes and nematodes [25]. Briefly, 3 g of feces were emulsified in 42 mL of water, stirred shortly, and passed through a 150 µm mesh. The filtered solution was collected into two 15 mL tubes and centrifuged at 2500 rpm for 10 min. The supernatant was discarded and 10 mL of saturated NaCl solution (ρ = 1.2 g/cm^3^) was added to each tube. After 2 min, aliquots of 300 µL were taken and observed in a McMaster chamber under a light microscope (4−10×) (Leica DM 2500, Barcelona, Spain).

The existence of eggs of trematodes in feces was investigated by means of the sedimentation probe [1]. Five grams of feces were blended with water, filtered through a 150 µm sieve, and passed to a 1 L conic cup. After decanting three times for 15 min, the content was reduced to 50 mL. Finally, aliquots of 300 µL were collected to fill a McMaster chamber, and then observed under an optical microscope (4−10×) (Leica DM 2500).

### 2.3. Isolation of Fungi from the Feces

By means of the flotation test, captive animals passing eggs of strongyles in their feces were identified. One gram of each fecal sample was placed onto a Petri dish containing water agar with chloramphenicol (WA) and incubated at 25 °C for 15 days [26]. Four replicates were considered for each sample.

Once fungal growth was recorded, fungal isolates were subcultured twice in malt extract agar (MEA; Drogallega, A Coruña, Spain) and corn meal agar (CMA, Sigma, MO, USA) for purification and subsequent identification, following standard protocols [27,28].

Monosporic cultures were obtained on potato glucose agar (PGA, Drogallega) for morphometric and cultural characterization. In some cases, subcultures were made on wheat extract agar with chloramphenicol (AT). Plates were incubated at 18–22 °C in the dark.

### 2.4. Identification of Fungal Species

The microscopic characterization of the fungal isolates consisted of measurements of 40 conidia, conidiophores, spores/chlamydospores, and sporanges by using an optical microscope (Olympus CX23LEDRFS1, Ashburton, New Zealand) equipped with a digital camera. Measurements were performed with an eyepiece micrometer scale. Identification of the fungal isolates was based on morphological features from pure cultures fungi, by means of keys and species descriptions [29,30,31,32,33].

### 2.5. Obtaining Parasites

Feces of cattle and horses with previous records of infection by parasites were collected and analyzed by using coprological probes. After the observation of eggs of *Calicophoron daubneyi* (gastric fluke) in bovine feces by using the sedimentation test, eggs were concentrated to 800 eggs/mL.

By applying the flotation probe, eggs of roundworms (*Parascaris equorum*) were identified in the feces of horses, then purified [21], and finally kept at a concentration of 800 eggs/mL.

### 2.6. Parasiticide Activity Testing Assays

Two assays were developed by using CMA plates. For each fungus isolated, two sets of plates were prepared; Set 1 received 400 eggs of *C. daubneyi*, and Set 2 received 400 eggs of *P. equorum*. Ten replicates were carried out for each fungus and parasite.

Ten plates without fungi were provided with 400 eggs of *C. daubneyi* as controls, and the same was done with eggs of *P. equorum*.

### 2.7. Evaluation of the Fungal Parasiticide Activity

Twenty-two days after placing the parasites, the CMA plates were observed under an optical microscope (Leica DM2500) for recording the changes in the eggs in comparison with their respective controls. Assessment of fungal damage on eggs was carried out according to the following alterations [34]:

- Type 0: Eggs are viable and damage or alterations are not observed.
- Type 1: Hyphae attached to the eggshell but morphological damage was not provoked.
- Type 2: The eggshell and embryo show damage without penetration.
- Type 3: Fungal hyphae enter the egg, grow, and destroy the embryo.

## 3. Results

A total of 13 captive animals passing eggs of strongyles in their feces were detected by means of the flotation test, thus these fecal samples were cultured and subcultured in search of fungal species with activity against parasite eggs.

**Figure 1:**
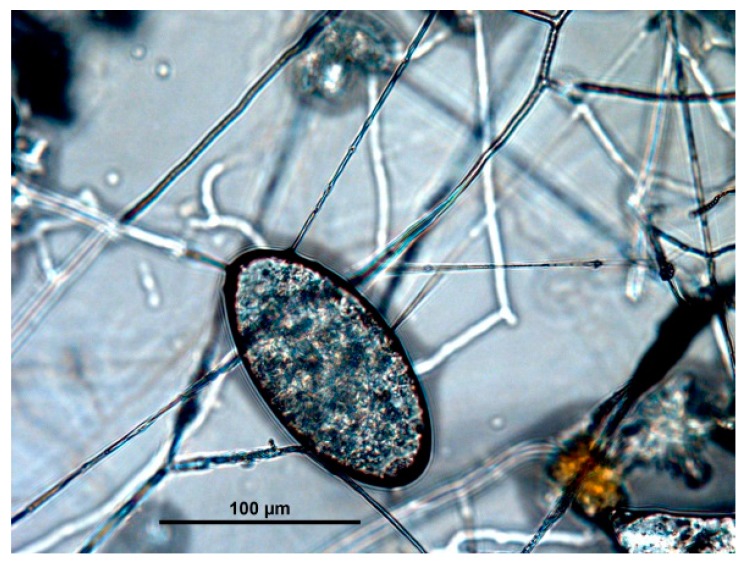
Hyphae of *Trichoderma* isolated from feces of captive wild animals developed in the presence of eggs of *C. daubneyi*.

In all the isolates, a mycelium was developed in the presence of eggs of *C. daubneyi* and *P. equorum*, and hyphae attached to the eggshell (type 1 activity) (Figure 1). Isolates identified as *Gliocladium* (two fecal samples) and *Penicillium* (*n* = 3) displayed only a type 1 ovicidal effect. The contact area between the hypha of fungi and the egg surface is smooth at the first stage. No damage of superficial structures of eggshell can be observed during this period. During the interaction with the egg, some hyphae of these fungi formed a lentiform penetration organ (*appresorium*) on the undeveloped egg surface. This is considered an important organ involved in the mechanism of penetration of the fungi through the solid eggshell.

**Figure 2:**
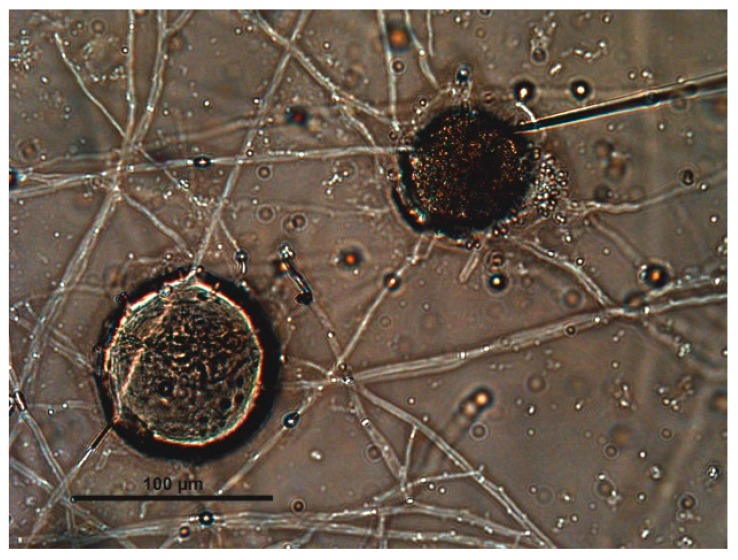
Eggshell of *Parascaris equorum* is penetrated by hyphae of *Mucor* spp.

Nine of the fungal isolates were also able to penetrate inside the eggshell after 6–10 days when the penetration organ (*haustorium*) started to damage the superficial structures of the chitin-protein layer of the envelope. As soon as the fungus has penetrated into the egg, it starts to form branches, and the formation of new hyphae was observed (Figure 2).

**Figure 3:**
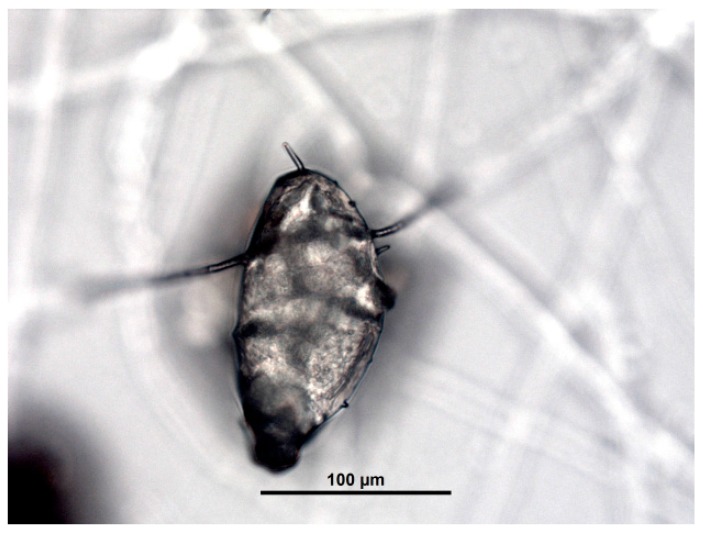
Destruction of the embryo inside egg of *C. dauneyi* by the action of *Trichoderma*.

**Figure 4:**
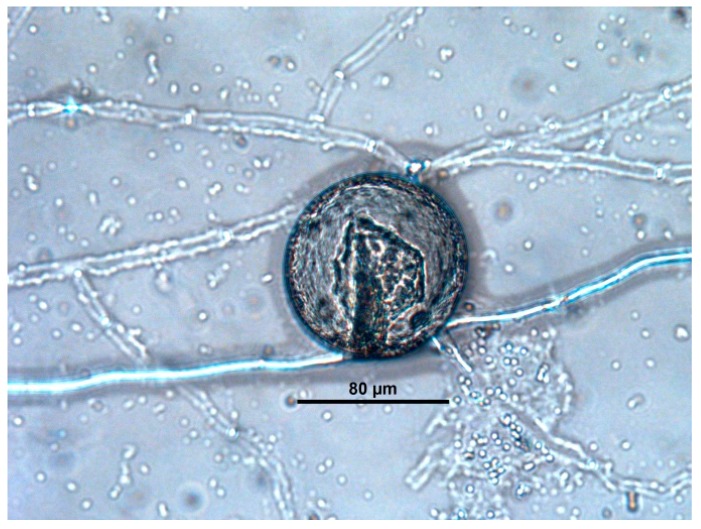
Destruction of the embryo inside egg of *P. equorum* exposed to *Verticillium*.

Finally, after attaching to the eggshells and penetrating them, the interior was colonized and, the inner embryo was destroyed (Figure 3 and Figure 4), so this ovicidal activity was classified as type 3. The consumption stage of the process begins here. The branching fungus starts to gradually liquidate the egg contents irrespective of the developmental stage of the embryo. The layer of the eggshell is already deformed.

**Figure 5:**
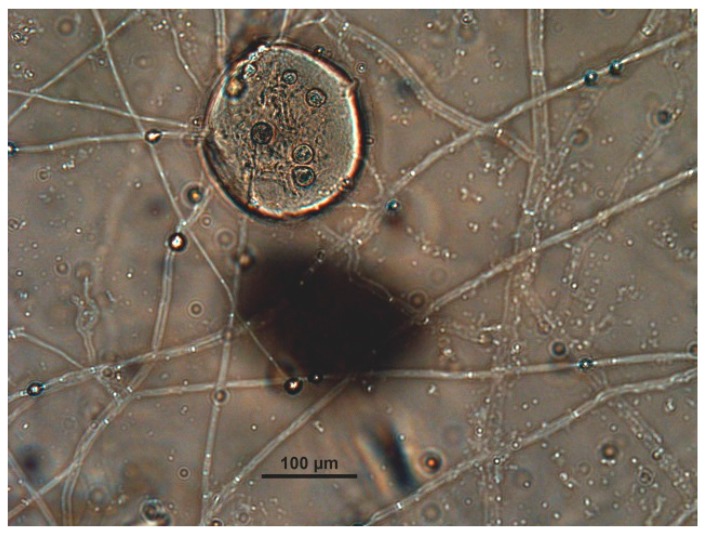
Chlamydospores of *Mucor* inside of an egg of *P. equorum*.

The last stage of the ovicidal process begins, when the ovicidal fungus leaves the liquidated and dead remnants of the nematode egg. In some cases, spores were also observed within the eggs (Figure 5). These isolates were identified as *Fusarium*, *Lecanicillium*, *Mucor*, *Trichoderma*, and *Verticillium*.

As summarized in Table 1, the number of fungal species with ovicidal activity in each of the fecal samples ranged between 1 and 2, whereas the most abundant predaceous fungi were, in four samples, *Trichoderma* and *Verticillium*, in three samples, *Fusarium*, *Mucor*, and *Penicillium*, *Gliocladium* in two samples, and *Lecanicilium* only in one.

No morphological differences regarding the effect the soil fungi developed have been recorded.

## 4. Discussion

The presence of soil fungi antagonists of egg parasites in fecal samples of wild captive animals was investigated. Formerly, only eggs of strongyles were detected in their feces. After culturing these fecal samples, seven isolates with ovicidal activity were obtained. Two of them, identified to genus level as *Gliocladium* and *Penicillium*, were able to adhere to eggshell only and therefore classified as type 1 ovicidal fungi. These are specimens found frequently in soil samples [35,36], and there is no available information concerning their effect on the eggs of helminths infecting animals. Some investigations reported their usefulness as a biocontrol agent against plant pathogens as *Rhizoctonia solani*, *Phytium ultimum*, and *Meloydogine incognita* [37,38,39].

Five fungal specimens isolated from the feces of the captive animals were identified to the genus level as *Fusarium*, *Lecanicillium*, *Mucor*, *Trichoderma*, and *Verticillium*. When eggs of the gastric fluke *Calicophoron daubneyi* and the roundworm *Parascaris equorum* were exposed to these fungi, it was observed that hyphae attached to the eggshells and penetrated and destroyed the inner embryo, so they were classified as type 3 ovicidal fungi. If the mechanical pressure was the main factor enabling the penetration of the hypha through the eggshell, then the fungi should be fixed to the egg surface in such a way that it would be able to develop a high pressure on the structure of eggshell, particularly on the mechanically resistant chitin–protein complex of the chitinous layer of *Ascaris lumbricoides* eggs. The penetration organ physically damages the eggshell, though specific enzymes may be involved. The damaged ascarosid layer no longer performs its role of the osmotic barrier. The embryo, if it has not yet been infected by the fungus, can be killed by the injurious substances from the outer environment, which can freely diffuse into the egg [34].

Prior investigations indicated the antagonistic activity of several fungal species belonging to the genera *Fusarium* and *Trichoderma* on eggs of the roundworm *Toxocara canis* [40,41,42]. The ovicidal activity of *Pochonia chlamydosporia* (formerly *Verticillium chlamydosporium*) has been widely reported on eggs of trematodes (*Echinostoma paraensei*, *Fasciola hepatica*) and ascarids (*T. canis*, *P. equorum*) [19,20,43].

Transmission of many parasites affecting animals occurs in the soil, where the infectious agents develop part of their life-cycle. This enhances the importance to control the numbers of infective stages of parasites, especially when animals are maintained always in the same paddocks, where the continuous shedding of eggs favors the accumulation of infective stages [5].

Different actions have been suggested to minimize the risk of infection in grazing animals, such as the rotation of pastures, the alternation of animal species pasturing in the same paddock, the manual collection of manure or drainage [44,45]. Nevertheless, these procedures can not be applied in different regimes, as occurs in zoological parks, and the number of dewormings per year is frequently increased to eliminate parasitic infections in the animals. As a consequence, selection of parasite strains resistant to different chemical compounds [46].

Nematodes in the soil are exposed to many organisms such as bacteria, viruses, fungi, predatory nematodes, and mites, and some have the ability to parasite and destroy them [47]. These beneficial organisms are called biological agents, which should be highly antagonistic to parasites, selective in their activity (acting on parasites but not on crop plants or higher animals), to growth on artificial media at suitable pH and temperature ranges, and easy to formulate in a working way [48]. In the current research, five of the isolated fungi developed in plates containing a medium composed of corn meal agar and a type 3 effect on eggs of *C. daubneyi* and *P. equorum* was recorded. No pathogen effects have been reported after the administration of *M. circinelloides*, *D. flagrans*, or *Pochonia chlamydosporia* to sheep, cattle, and domestic and wild horses [2,5,7,49,50]. In recent years, the formulation of fungi in pelleted feed by adding mycelium or spores of *D. flagrans* and *M. circinelloides* provided successful results in terms of preventing infection by helminths in horses, enhancing thus their distribution as biocontrol agents [2,51].

Parasitic stages present in soil can also affect humans. Soil-transmitted helminth infections caused by ascarids, such as *Toxocara canis*, *Ascaris suum*, *Toxascaris leonina*, and *Baylisascaris procyonis*, and trichurids are transmitted to humans through the accidental ingestion of eggs containing a second stage larva inside [21,42]. Larvae of ancylostomids can penetrate through the skin and especially affect people enjoying recreational locations with, for example, sandy areas or recreational surfaces in parks [52,53]. Parasiticide fungi could be distributed by spraying them into aqueous solutions, providing a useful tool for lessening the risk of infection to children playing in those places or adults taking a sunbath.

Our results demonstrated the existence of saprophytic fungi with ovicidal activity in the feces of captive animals from the “Marcelle Natureza” zoological park (Lugo, NW Spain), and a similar variety of predaceous fungi was observed in all samples examined. Most of the former surveys conducted on fecal samples collected from domestic livestock have been focused on the finding predaceous fungi with larvicidal activity only [4,13,14,54].

In the present investigation, the presence of fungal specimens in feces indicates that they can survive the gastrointestinal tract, and the observation of an ovicidal type 3 effect in five of the isolates demonstrates that fungi retained their biological activity on the eggs of helminths, confirming thus their potential as biological control agents against helminths transmitted through eggs. Their impact on the eggs of strongyle nematodes remains unknown because, according to the weather and/or season, the development of a mobile phase (larva) inside might be slower than the time required for the fungi to develop their ovicidal activity. Further studies are in progress to elucidate this issue.

## Figures and Tables

**Table 1:** Isolation of predaceous fungi with ovicidal activity against eggs of the helminths *Calicophoron daubneyi* and *Parascaris equorum*, in feces of wild captive animals (“Marcelle Natureza” zoological park, NW Spain). WA: water agar. MEA: malt extract agar. CMA: corn meal agar. PGA: potato glucose agar. AT: wheat extract agar with chloramphenicol.

Captive Animals			Fungal Isolation			
Common Name	Scientific Name	Parasites Diagnosed	Culture	Subculture 1	Subculture 2	Genera Identified
Coati	*Nasua nasua*	Nematodes: Strongyles	WA	MEA		*Trichoderma*
				CMA		*Trichoderma*
						*Verticillium*
Raccoon	*Procyon lottor*	Nematodes: Strongyles	WA	MEA		*Mucor*
				CMA		*Mucor*
Eurasian lynx	*Lynx lynx*	Nematodes: Strongyles	WA	MEA	PGA	*Fusarium*
				CMA		*Gliocladium*
Brown bear	*Ursus arctos*	Nematodes: Strongyles	WA	MEA		*Trichoderma*
				CMA		*Trichoderma*
Goat	*Capra hircus* spp.	Coccidia	WA	MEA	PGA	*Verticillium*
		Nematodes: Strongyles		CMA		*Verticillium*
Mouflon	*Ovis musimon*	Coccidia	WA	MEA		*Fusarium*
						*Penicillium*
		Nematodes: Strongyles		CMA		*Fusarium*
Gazelle	*Gazella cuvieri*	Coccidia	WA	MEA		*Mucor*
		Nematodes: Strongyles		CMA		*Mucor*
						*Penicillium*
Axis	*Axis axis*	Nematodes: Strongyles	WA/AT	MEA		*Verticillium*
						*Lecanicillium*
				CMA		*Verticillium*
						*Lecanicillium*
Bison	*Bison bison*	Coccidia	WA	MEA		*Trichoderma*
		Nematodes: Strongyles		CMA		*Trichoderma*
Dromedary	*Camelus dromedarius*	Nematodes: Strongyles	WA	MEA	PGA	*Trichoderma*
				CMA		*Trichoderma*
						*Verticillium*
Guanaco	*Lama guanicoe*	Coccidia	WA	MEA	PGA	*Gliocladium*
		Nematodes: Strongyles		CMA		*Gliocladium*
Falabella	*Equus caballus*	Coccidia	WA	MEA		*Fusarium*
				CMA		*Fusarium*
						*Penicillium*
Wallaby	*Macropus rufogriseus*	-	WA	MEA		*Mucor*
				CMA		(Sordariaceae)
